# Effect of tryptase on mouse brain microvascular endothelial cells via protease-activated receptor 2

**DOI:** 10.1186/s12974-018-1287-1

**Published:** 2018-08-31

**Authors:** Qin Zhou, Yi-wei Wang, Peng-fei Ni, Yi-nan Chen, Hong-quan Dong, Yan-ning Qian

**Affiliations:** Department of Anesthesiology, The First Affiliated Hospital of Nanjing Medical University, Nanjing, 210029 Jiangsu People’s Republic of China

**Keywords:** Brain microvascular endothelial cells, Tryptase, Protease-activated receptor 2 (PAR-2), MAPK, NF-kappa B

## Abstract

**Background:**

Mast cells (MCs), the ‘first responders’ in brain injury, are able to disrupt the blood–brain barrier (BBB), but the underlying mechanism is not well understood. Tryptase is the most abundant MC secretory product. Protease-activated receptor 2 (PAR-2) has been identified as a specific receptor for tryptase, which is abundantly expressed in brain microvascular endothelial cells. The BBB comprises brain microvascular endothelial cells that display specialised molecular properties essential for BBB function and integrity. Therefore, the purpose of the present study was to investigate the effects of tryptase on mouse brain microvascular endothelial cell line bEnd3 and its potential mechanisms of action.

**Methods:**

Induction of mouse brain microvascular endothelial cell activation by tryptase was examined. Then, mouse brain microvascular endothelial cells were pretreated with a PAR-2 antagonist and stimulated with tryptase. Cellular activation, proinflammatory cytokine production, expression of PAR-2, Toll-like receptors (TLRs) and mitogen-activated protein kinases (MAPK), nuclear factor kappa B (NF-kappa B) phosphorylation were assessed.

**Results:**

Tryptase upregulated the production of VCAM-1, MMPs (MMP9 and MMP2), TLR4 and TNF-α and downregulated the expression of the tight junction proteins occludin and claudin-5 in mouse brain microvascular endothelial cell. Among the MAPK and NF-kappa B pathway, ERK and NF-kappa B were activated by tryptase. All of these effects could be eliminated by the PAR-2 inhibitor.

**Conclusion:**

Based on our findings, we conclude that tryptase can trigger brain microvascular endothelial cell activation and proinflammatory mediator release. These findings may further clarify the involvement and mechanism of tryptase in BBB disruption.

## Background

Mast cells (MCs) are multifunctional immune cells that can maintain and regulate immune function, best known for the important role in allergic inflammation [1]. There is increasing evidence showing that MC degranulation in the brain is involved in central nervous system (CNS) inflammatory processes [2–4]. However, the mechanisms underlying how mast cells disrupt the BBB are unclear.

Tryptase is the major secretory protein of mast cell degranulation [5]. Upon activation, MCs secrete tryptase, which can contribute to microvascular leakage in guinea pigs and induce the recruitment of inflammatory cells in the peritonea of mice [6, 7]. It can also stimulate peripheral mononuclear cells to release interleukin-6 (IL-6) and tumour necrosis factor-alpha [8]. In vitro, tryptase can contribute to microglia and astrocyte activation and release of proinflammatory mediators via mitogen-activated protein kinases (MAPK) and nuclear factor kappa B (NF-kappa B) [9, 10]. These observations indicate that tryptase has a vital role in MC-associated inflammation.

Recent studies have found that PAR-2 is widely expressed in the brain, including the BBB and cerebral microvascular endothelial cells. Furthermore, the activation of PAR-2 is associated with neuroinflammation and neurodegenerative diseases [11, 12]. Reports have showed that PAR-2 activation can contribute to microglial activation, which induces neuronal cell death, and activation of PAR-2 destroys the BBB during cerebral damage [13, 14].

Cerebral microvascular endothelial cells are the major components of the BBB and tight junction protein (TJP) network produced by endothelial cells to maintain the integrity of the BBB [11, 15]. A report showed TJP degradation increases endothelial cell permeability, destroying BBB integrity [16]. Cerebral microvascular endothelial cells can also express matrix metalloproteinases (MMPs), which are markers of inflammation. Matrix metalloproteinase 2 (MMP2) and MMP9 can degrade TJPs, disrupting the integrity of the BBB [17].

We previously demonstrated that MC degranulation can disrupt the BBB [4]. We also found that the supernatant from activated MCs can induce mouse brain microvascular endothelial cell activation and promote the secretion of the inflammatory cytokines TNF-α and IL-6. However, the effect of MC tryptase on mouse brain microvascular endothelial cell has not yet been studied. In the present study, we investigated the possibility that tryptase could trigger mouse brain microvascular endothelial cell activation through PAR-2.

## Methods

### Reagents

Dulbecco’s modified Eagle’s medium (DMEM), foetal bovine serum (FBS) and 0.25% Trypsin–EDTA solution were purchased from Gibco-BRL (Grand Island, NY, USA). Tryptase was purchased from Sigma-Aldrich (St. Louis, MO, USA), and it is the human lung tryptase, which is a neutral serine protease and the predominant protein in mast cell granules. PAR-2 inhibitor FSLLRY-NH2 (FS) was synthesised by CL Bio-Scientific Inc. (Xi An, China). CCK-8, RIPA buffer and the BCA kit were purchased from Beyotime (Shanghai, China). Rabbit anti-PAR-2 polyclonal antibody and fluoroshield mounting medium with 4′,6-diami-dino-2-phenylindole (DAPI) were purchased from Abcam (Hongkong, China). Anti-TLR4 monoclonal antibody, anti-VCAM-1 antibody (EPR5 047) and anti-occludin antibody (EPR8208) were purchased from Abcam (Hongkong, China). Anti-GAPDH antibody was purchased from Bioworld Technology, Inc. (USA). Anti-p44/42 MAPK monoclonal antibody (extracellular regulated protein kinases, ERK), anti-Phospho-p44/42 monoclonal antibody (phosphoERK) and NF-kappa B were purchased from Cell Signaling (Beverly, MA, USA).Anti-rabbit and anti-mouse secondary antibodies were all purchased from Jackson Immuno Research Laboratories Inc. (Boston, MA, USA).

### Cell cultures

The mouse brain microvascular endothelial cell line bEnd.3 was purchased from Shanghai Bioleaf (Shanghai, China). bEnd.3 cells were cultured in DMEM (Thermo Fisher Scientific, Waltham, MA,USA) containing 10% foetal bovine serum (FBS), 100 μg/mL penicillin and 100 μg/mL streptomycin (pH = 7.2–7.4) [16, 18]. The cells were seeded on poly-d-lysine pre-coated cell culture flasks and cultured at 37 °C in a humidified atmosphere of 5% CO2/95% air. Two-milliliter medium was given to the cells. The medium was replaced every 2 to 3 days after seeding. They were passaged at the same density until the cell confluence reached 80%. The cells were starved overnight and then subjected to treatments and were pretreated with FS (400 μM) for 30 min before tryptase (1 μg/mL) stimulation, and the volume of tryptase was 1 μL (the concentration of tryptase is 2 μg/μL). This experiment consists of four groups (control group, tryptase group, FS group, tryptase + FS group). We dissolved tryptase and FS in PBS, so we also added the same volume of the PBS in the control group.

### Cell viability

In order to measure the toxicity of tryptase and FS to mouse brain microvascular endothelial cell, experimental groups were divided into eight groups receiving different concentrations of tryptase (0.001, 0.01, 0.1, 1, and 10 μg/mL) [9, 10], FS (400 μM, 800 μM) [5, 9, 10] and control group. We added the same volume of the PBS to the control group. Briefly, mouse brain microvascular endothelial cell were seeded into 96-well plates at a density of 5000 cells/well, and 100 μL of the medium was added to each well. Twenty-four hours after the incubation, the medium was discarded and 100 μL of fresh medium was added to the normal control groups. Media containing tryptase and FS of different concentrations were added to experimental groups. After 24 h, the WST-8 dye (Water-soluble tetrazoliuM-8,Cell Counting Kit-8, CCK-8 main components) was added to each well, and the cells were then incubated at 37 °C for 2 h. The absorbance was determined at 450 nm using a microplate reader.

### Western blotting

Cellular proteins were extracted by RIPA buffer after mouse brain microvascular endothelial cells were stimulated by drugs for 24 h. A BCA kit was used to measure the protein concentration in the lysate supernatant. Cell extract proteins (50 μg) were denatured with sodium dodecyl sulfate (SDS) sample buffer and separated using 10% SDS–polyacrylamide gel electrophoresis (PAGE). The proteins were transferred to a polyvinylidene fluoride (PVDF) microporous membrane (Millipore, Bedford, MA, USA), which was then blocked with 5% skim milk for 1 h at room temperature. The membrane was incubated with primary antibody overnight at 4 °C. The following primary antibodies were used: rabbit monoclonal anti-PAR-2 (Abcam, EPR13675, 1:1000), rabbit monoclonal anti-VCAM-1 (Abcam, EPR5047, ab134047, 1:2000), rabbit monoclonal anti-GAPDH (Bioworld Technology Inc., Ap0063, 1:1000), rabbit monoclonal anti-MMP-9 (Abcam, 1:500), rabbit monoclonal anti-MMP-2 (Abcam, 1:1000), rabbit monoclonal anti-occludin (Abcam, EPR8208, ab167161, 1:50,000), mouse monoclonal anti-claudin-5 (Invitrogen, 1:500), and mouse monoclonal anti-TLR4 (Abcam, 76B357.1, ab22048, 4 μg/ml). After adding the anti-rabbit or anti-mouse secondary antibody (Jackson Immuno Research Laboratories INC. number: 122825 and 122627, 1:1000) for 1 h, protein bands on the membranes were detected using an enhanced chemiluminescence kit. The relative density of the protein bands was obtained by densitometry using Image Lab software (Bio-Rad, Richmond, CA, USA) and quantified using NIH ImageJ software (Bethesda, MD, USA).

### Immunofluorescence

To examine the activation of the mouse brain microvascular endothelial cell and the expression of PAR-2, cells were fixed with 4% paraformaldehyde for 30 min. Non-specific binding was blocked by incubating cells in a 5% BSA and 0.1% Triton X-100 solution for 1 h at room temperature. Mouse brain microvascular endothelial cells were incubated with rabbit anti-VCAM-1 polyclonal antibody (1:200) or rabbit anti-PAR-2 polyclonal antibody (1:100) in the blocking solution overnight at 4 °C. After three washes with PBS, mouse brain microvascular endothelial cells were incubated with the corresponding FITC-conjugated goat anti-rabbit IgG (1:200) for 2 h at room temperature. Nuclei were stained with DAPI. Fluorescent images were acquired using a confocal microscope.

### Enzyme-linked immunosorbent assay (ELISA)

The amount of TNF-α in the culture medium was measured using a commercial ELISA kit from R&D Systems (Minneapolis, MN, USA) according to the manufacturer’s instructions.

### Real-time PCR

Total RNA was extracted from mouse brain microvascular endothelial cell cultures using Trizol Reagent (Invitrogen), and reverse transcription was performed from 1 μg of total RNA for each sample using the Transcription First Strand cDNA Synthesis Kits (Roche) according to the manufacturer’s instructions. Real-time PCR amplification was performed using the StepOne Plus Real-Time PCR System (Applied Biosystems) with the SYBR Green master mix (Applied Biosystems, Foster City, CA) in a final volume of 10 μg that contained 1 μg of cDNA template from each sample. Primers used were as follows: mouse GAPDH forward, 5′-AACTTTGGCATTGTGGAAGG-3′, and reverse, 5′-GGATGCAGGG- ATGATGTTCT-3′; mouse TNF-α forward, 5′-GACGTGGAACTGGCA- GAAGAG-3′; and reverse, 5′-TTGGTGGTTTGTGAGTGTGAG-3′. The cycling conditions were 95 °C for 10 min, followed by 40 cycles of 95 °C for 15 s and 60 °C for 1 min. The relative mRNA values were normalised to GAPDH gene control values and calculated using the comparative cycle threshold (ΔΔCt) method.

### Statistical analysis

All values are expressed as the mean ± S.E.M. Statistical analysis was carried out with GraphPad Prism 5 software (version 5.01, GraphPad Software, San Diego, CA). Data were analysed with one-way ANOVA, followed by Newman–Keuls post hoc testing where appropriate. Statistical significance was accepted at *p* < 0.05.

## Results

### Effects of tryptase and FS on cell viability in mouse brain microvascular endothelial cell

**Fig. 1:**
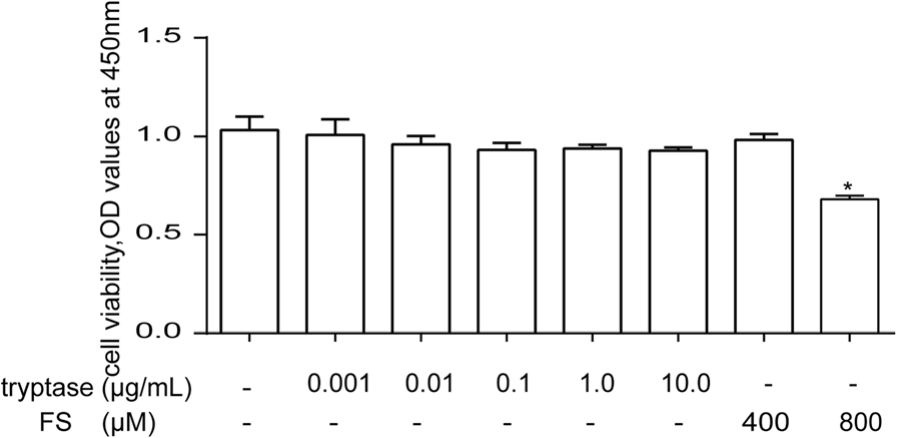
The effects of tryptase and FS on cell viability in mouse brain microvascular endothelial cell. Mouse brain microvascular endothelial cells were exposed to different concentrations of tryptase (0.001, 0.01, 0.1, 1, and 10 μg/mL) and FS (400 μM, 800 μM) and control group for 24 h. Cell viability was determined using a colorimetric method. *P < 0.05 versus the control group. Each data point represents the mean ± S.E.M of at least three separate experiments

The Cell Counting Kit-8 (CCK-8) assay was used to evaluate the toxic effects of tryptase and FS (a PAR-2 inhibitor) on mouse brain microvascular endothelial cell. The mouse brain microvascular endothelial cells were divided into eight groups, including different concentrations of tryptase (0.001, 0.01, 0.1, 1 and 10 μg/mL), FS (400 μM, 800 μM) and control group. Then cell viability was measured by the CCK-8 assay after incubating 24 h. Our results indicate that tryptase (1 μg /mL) and 400 uM FS exerted no obvious toxic effects on mouse brain microvascular endothelial cell (Fig. 1).

### Tryptase can promote the expression of PAR-2 on mouse brain microvascular endothelial cell

**Fig. 2:**
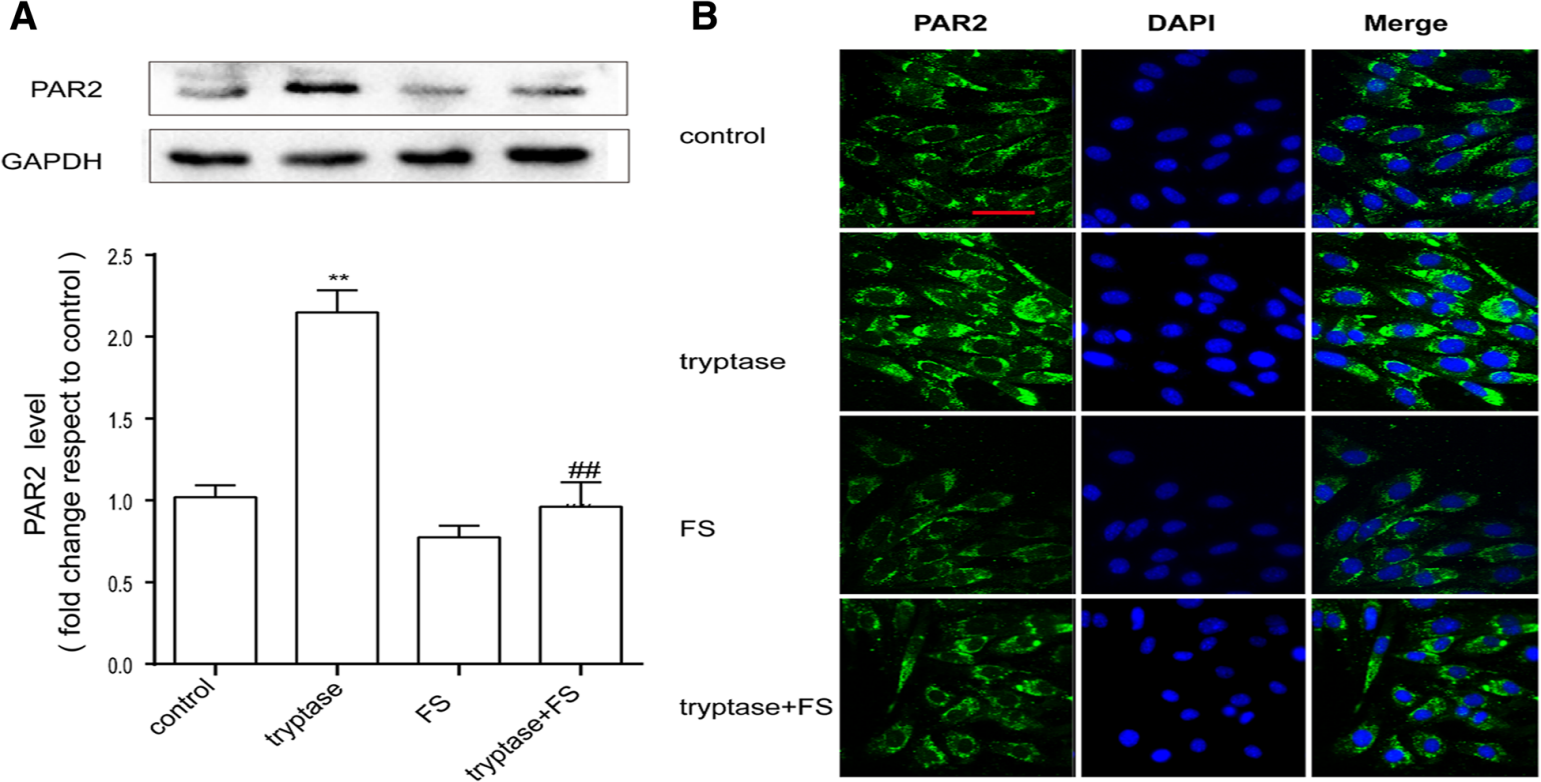
Tryptase can increase PAR-2 levels. Mouse brain microvascular endothelial cells were pre-treated with FS at 400 μM for 30 min and then stimulated with tryptase at 1 μg/mL for 24 h. **a** Western blotting analysis of PAR-2 levels in which the levels of PAR-2 were quantified and normalised to their respective GAPDH levels. Each value was then expressed relative to the control, which was set to 1 (*n* = 3). **b** Cells were stained with anti-PAR-2 antibody and unregulated PAR2-immunopositive expression (green) on the activated bEnd.3 cells was observed using confocal scanning. The blue staining represents DAPI. Scale bar: 25 μm. **P < 0.01 versus the control group. ^##^*P* < 0.01 versus the tryptase group. The data are presented as the mean ± S.E.M of at three separate experiments

As tryptase is the natural agonist of PAR-2, we examined the influence of tryptase on the expression of PAR-2 by western blotting and immunofluorescence. As shown in Fig. 2a, b, tryptase could upregulate the protein level of PAR-2. However, pre-treatment with FS (a PAR-2 inhibitor) resulted in decreased expression of PAR-2 compared with the control group.

### Tryptase upregulates the production of VCAM-1 via PAR-2

**Fig. 3:**
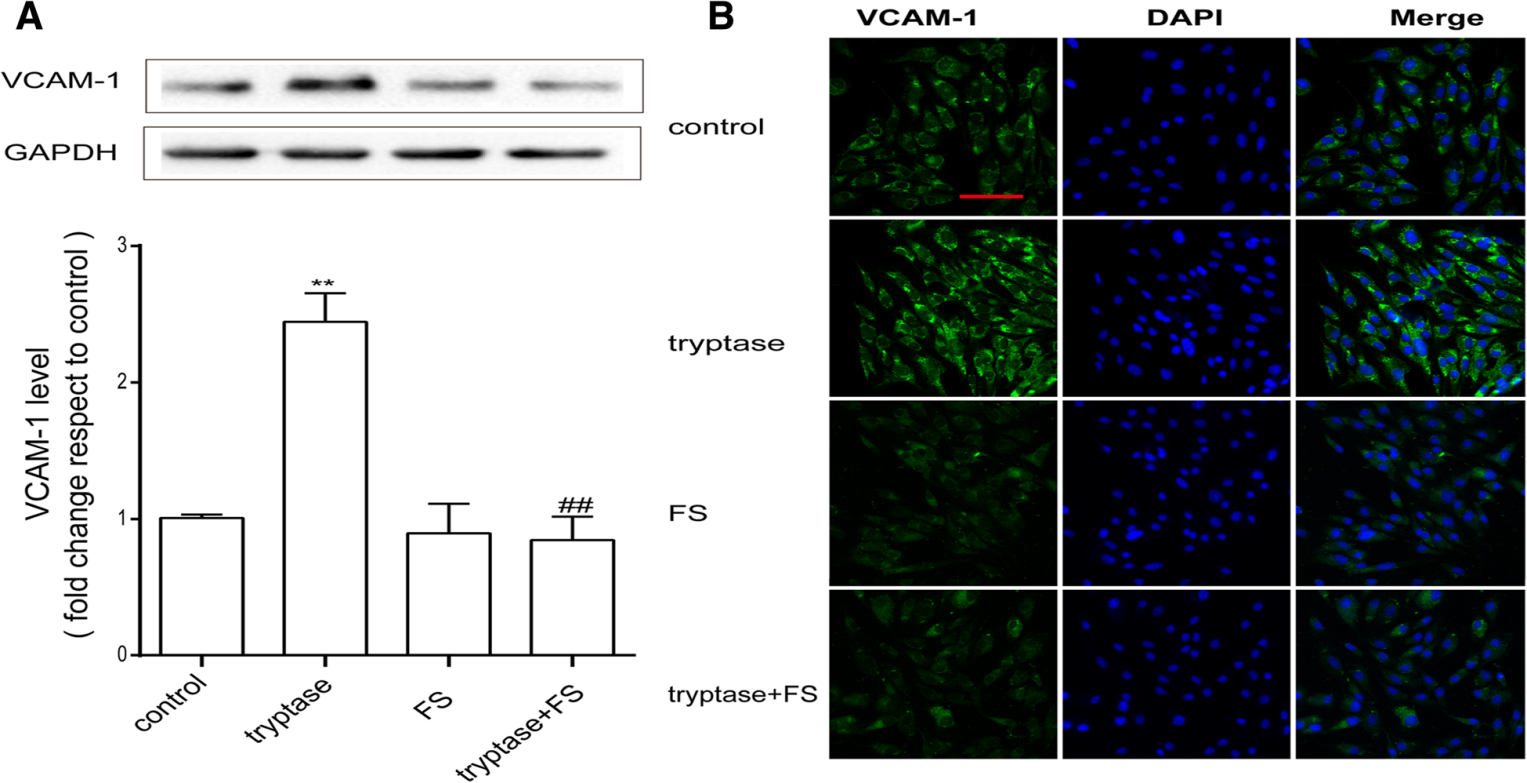
Tryptase upregulates the production of VCAM-1 via PAR-2. Mouse brain microvascular endothelial cell were pre-treated with FS at 400 μM for 30 min and then stimulated with tryptase at 1 μg/mL for 24 h. **a** Western blotting analysis of VCAM-1 levels in which the levels of VCAM-1 were quantified and normalised to their respective GAPDH levels. Each value was then expressed relative to the control, which was set to 1 (*n* = 3). **b** The cells were stained with anti-VCAM-1 antibody, and upregulated VCAM-1-immunopositive expression (green) on the activated mouse brain microvascular endothelial cell was observed using confocal scanning. The blue staining represents DAPI. Scale bar: 50 μm. **P < 0.01 versus the control group. ^##^*P* < 0.01 versus the tryptase group. The data are presented as the mean ± S.E.M of at three separate experiments

To confirm that mouse brain microvascular endothelial cell activation was induced by tryptase, we used western blotting and immunofluorescence to examine the expression of the adhesion molecule VCAM-1. Cells were pretreated with FS (400 μM) for 30 min and then stimulated with tryptase (1 μg/ml) for 24 h. Western blotting results showed that tryptase significantly upregulated the expression of VCAM-1 (Fig. 3a). Immunofluorescence results also showed that compared with the control group, tryptase significantly increased VCAM-1 levels (Fig. 3b). However, pre-incubation with FS could reverse this phenomenon.

### Effect of tryptase on the production of occludin and claudin-5

**Fig. 4:**
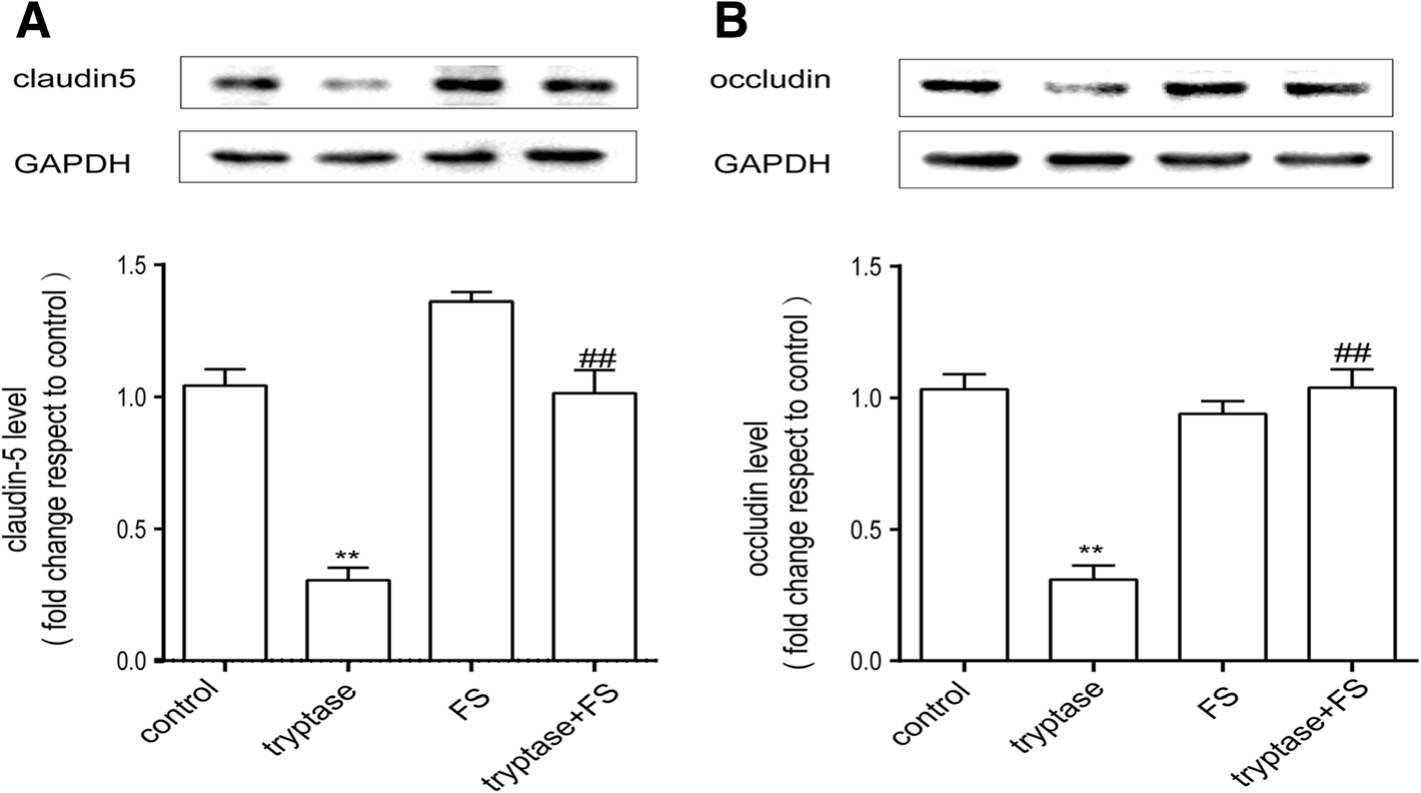
Tryptase can downregulate the production of tight-junction proteins. Mouse brain microvascular endothelial cells were accordingly pretreated with FS at 400 μM for 30 min and then stimulated with tryptase at 1 μg/ml for 24 h. **a**, **b** Western blotting analysis of claudin-5 and occludin levels, which were quantified and normalised to the respective GAPDH. Each value was then expressed relative to the control, which was set to 1 (*n* = 3). **P < 0.01 versus the control group. ^##^*P* < 0.01 versus the tryptase group. The data are presented as the mean ± S.E.M of at three separate experiments

We used western blotting to examine the expression of the tight junction proteins occludin and claudin-5, which are associated with BBB integrity. As shown in Fig. 4, following incubation with tryptase at 1 μg/ml for 24 h, the production of proteins from mouse brain microvascular endothelial cell clearly decreased compared with the control group. Pre-incubation with FS (400 μM) for 30 min could eliminate these effects.

### Tryptase can upregulate the production of MMP2 and MMP9

**Fig. 5:**
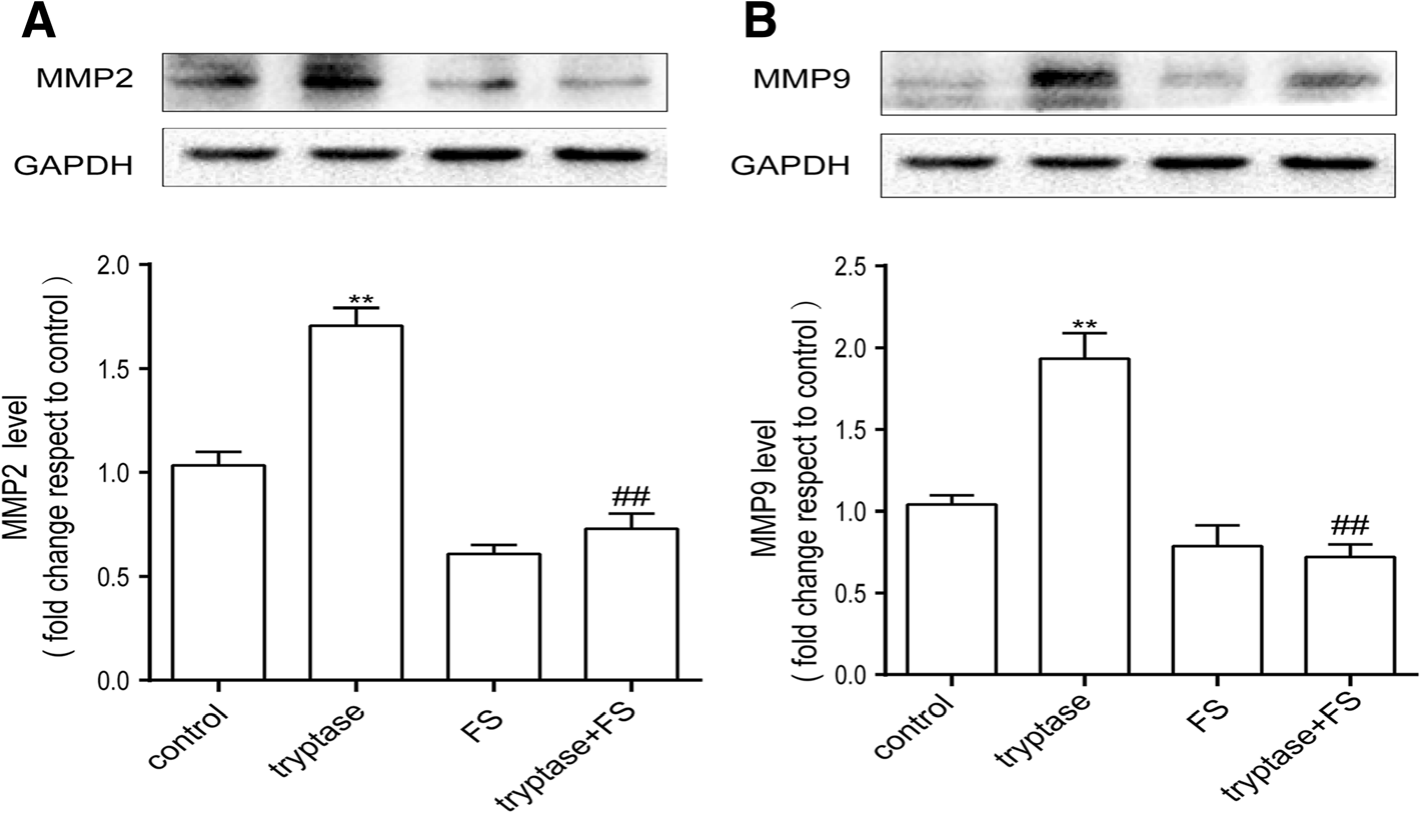
Tryptase could upregulate the production of MMP-9 and MMP-2. Mouse brain microvascular endothelial cell were accordingly pretreated with FS at 400 μM for 30 min and then stimulated with tryptase at 1 μg/ml for 24 h. **a**, **b** Western blotting analysis of MMP-9 and MMP-2 levels, which were quantified and normalised to their respective GAPDH levels. Each value was then expressed relative to the control, which was set to 1 (*n* = 3). **P < 0.01 versus the control group. ^##^*P* < 0.05 versus the tryptase group. The data are presented as the mean ± S.E.M of at three separate experiments

MMPs regulate the expression of occludin family members and are markers of inflammation. Therefore, we next examined the effect of tryptase on the production of MMPs, especially MMP-9 and MMP-2. As shown in Fig. 5, after stimulation with tryptase at 1 μg/ml for 24 h, MMP-9 and MMP-2 levels increased compared with the control group. Pre-incubation with FS (400 μM) for 30 min could reverse this phenomenon.

### Tryptase activated MAPK and NF-kappa B pathway and promoted cytokine production via PAR-2

**Fig. 6:**
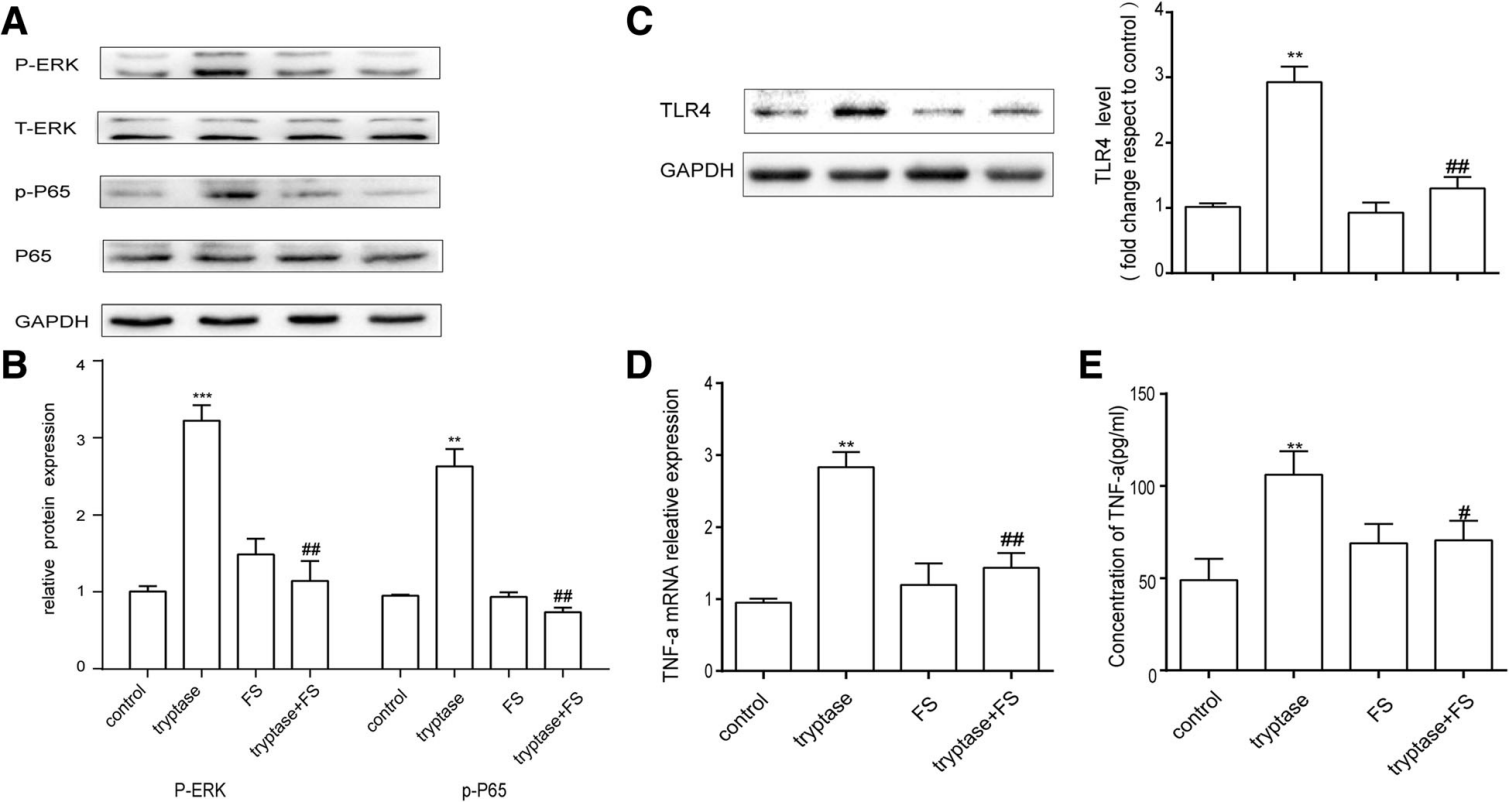
Tryptase activated MAPK and NF-kappa B pathway. FS (400 μM) incubated mouse brain microvascular endothelial cell for 30 min, then tryptase(1 μg/ml) treated for another 30 min. **a** Western blotting analysis of signalling pathway-associated protein expression. Tryptase activated the ERK and NF-kappa B p65, but FS suppressed the phenomenon. **b** Expression levels of ERK and NF-kappa B p65 were measured by western blot. **c** Western blotting analysis of TLR4 levels, which were quantified and normalised to their respective GAPDH levels. **d** RT-PCR analysis of the relative expression of TNF-α mRNA. **e** Concentration of TNF-α measured by ELISA. Each value was then expressed relative to the control, which was set to 1 (*n* = 3). ***P < 0.001, **P < 0.01 versus the control group. ^##^*P* < 0.01, ^#^*P* < 0.05 versus the tryptase group. The data are presented as the mean ± S.E.M of at three separate experiments

In order to further study the action of tryptase on mouse brain microvascular endothelial cells, we examined the effect of tryptase on phosphorylation of cell signalling molecules. Endothelial cells were pre-treated with PAR-2 antagonist FS(400 μM) for 30 min and then stimulated by tryptase (1 μg/ml) for another 30 min for examining phosphorylation of ERK and NF-kappa B p65. As shown in Fig. 6a, b, tryptase induced a high phosphorylation of ERK and NF-kappa B p65 compared with control group, which was inhibited by PAR-2 antagonist FS. To observe inflammation effects of tryptase, proinflammation factor TNF-α and TLR4 were determined, respectively. Figure 6c–e shows that tryptase could promote the expression of TNF-α and TLR4. FS counteracted this increase as well. These phenomenons show that tryptase activates ERK-MAPK and NF-kappa B p65 signalling pathway and promotes the inflammation mediator secretion via PAR-2.

## Discussion

The BBB maintains an optimum ionic composition of cerebrospinal fluid (CSF), which limits the entry of plasma components and supports the unique metabolic needs of the brain, providing a stable environment for proper brain function. Disruption of the BBB has been proven to be related to various neurological disorders, such as Alzheimer’s disease (AD), Parkinson’s disease (PD), and postoperative cognitive dysfunction (POCD) [19, 20]. In the early stages of the pathogenesis of acute cerebral ischaemia and cerebral haemorrhage, mast cells exhibit the rapid degranulation response in cerebral blood vessels and other parts of the CNS, releasing vasoactive and neuroactive synthetic medium in advance. The active medium acts on the basement membrane to disrupt the BBB and brain oedema, promoting the infiltration of cells and haemorrhage [2]. In addition, in acute cerebral ischaemia injury, brain mast cells increase in number, which prolongs the expression of endothelial cell adhesion molecules, destroys the BBB and recruits neutrophils, macrophages and other inflammatory cells into the centre of the injury [21, 22].Therefore, MCs play a vital role in the disruption of the BBB, but the details of how they work are still unknown. Thus, in the present work, we focused on bEnd.3 cells with an emphasis on their potential role in BBB integrity.

The current study demonstrated for the first time that stimulation of PAR-2 on bEnd.3 cells with tryptase triggered the activation of mouse brain microvascular endothelial cell and resulted in the degradation of occludin and claudin-5, increasing the production of the inflammatory mediators MMP-9, MMP-2 and TNF-α. The signalling pathway MAPK–NF-kappa B is associated with the tryptase-induced PAR-2 activation. However, FS could eliminate the effect of tryptase on the bEnd.3 cells. The result of CCK8 showed that the concentration of FS (800 μM) is toxic to bEnd.3 cells, so we chosed the concentration of FS (400 μM) to stimulate the cells.

VCAM-1 is a symptom of endothelial cell activation and is associated with mononuclear cell adhesion and immune cell transmigration [23]. Brain microvascular endothelial cells are important sources of VCAM-1. The reports demonstrated that cerebral endothelial cells could express VCAM-1 and contribute to lymphocyte adhesion and upregulation of VCAM-1 on endothelial cells could promote T cell transmigration [24, 25]. A considerable amount of evidence suggests the expression of VCAM-1 is linked to brain endothelial cell activation and immune cell transmigration. Like VCAM-1, matrix metalloproteinases (MMPs), especially MMP-2 and MMP-9, appear to also play an important role in the early disruption of the BBB [26, 27]. MMPs are markers of inflammation, and upon activation, MMPs can degrade most of the protein constituents of the neurovascular matrix, such as elastin, collagen, fibronectin, vitronectin, and gelatin [28]. Here, we found that tryptase increased VCAM-1 and MMP secretion by mouse brain microvascular endothelial cell. However, FS (PAR-2 antagonist) was able to block the secretion of VCAM-1, MMP-9 and MMP-2. PAR-2 was found to be expressed abundantly on brain microvascular endothelial cell, and PAR-2 activation in endothelial cell has been demonstrated to play a key modulatory role in diverse pathological conditions [11, 12].

Tryptase, a major MC secretory protein, is an endogenous peptide. Tryptase is involved in angiogenesis after they are secreted from activated MC granules and its proteolytic activities release matrix-associated growth factors or degrade extracellular matrix components associated with a critical step in the early stages of angiogenesis as well as during invasion and metastasis of tumour cells [29]. So tryptase is a novel angiogenesis factor, which also specifically activates PAR-2 [5]. In our study, we found that incubation with tryptase decreased the expression of claudin-5 and occludin in mouse brain microvascular endothelial cell. FS (a PAR-2 antagonist) diminished the effect of tryptase on claudin-5 and occludin expression, implying that the inhibition of claudin-5 and occludin by tryptase may at least partially be responsible for the activation of PAR-2. In the brain, tight junction proteins such as claudin-5 and occludin at the lateral apical membrane establish a high-resistance paracellular barrier to small hydrophilic molecules and ions. There is increasing evidence supporting a role for TJ-associated proteins in the permeability and integrity of the BBB [15, 16]. Reports have found that degradation of claudin-5 and occludin can increase the permeability and contribute to brain oedema in ischaemic stroke [30]. And claudin-5 regulates the permeability and integrity of the BBB by changing endothelial or epithelial cell permeability [31]. Our current observation suggests that moderate stimulation of PAR-2 on mouse brain microvascular endothelial cell may regulate tight junction proteins and supply a new option for the management of CNS functions.

TJP-occludin and claudin-5 produced by brain endothelial cells are good for the BBB, but brain endothelial cells also play an important role in regulating inflammatory responses at the BBB by releasing cytokines. Brain endothelial cells could synthesise CCL2 and CCL3 with significant upregulation after LPS activation [32]. Recent studies have found that activation of PAR-2 is also associated with inflammation [33, 34]. In type 2 diabetic mice, stimulation of PAR-2 can upregulate TNF-α expression [35]. MAPK and NF-kappa B signalling pathway are associated with the inflammation progress in endothelial cells [36]. Therefore, we examined the expression of TLR4 and TNF-α and MAPK and NF-kappa B signalling pathway-associated protein when PAR-2 on mouse brain microvascular endothelial cell was stimulated by tryptase. Our results were similar to those of previous reports that the stimulation of PAR-2 promotes secretion of the inflammatory cytokine TNF-α and upregulates the level of TLR4 and activates ERK and NF- kappa B p65. However, FS (a PAR-2 antagonist) could protect mouse brain microvascular endothelial cell by inhibiting TNF-α secretion and phosphorylation of ERK and NF-kappa B p65.

## Conclusion

In conclusion, to our knowledge, this is the first study to demonstrate the ability of mast cell tryptase to modulate mouse brain microvascular endothelial cell activation and inflammatory factor production via PAR-2. The possible intracellular signalling mechanisms are PAR-2–MAPK–NF-kappa B pathway. The results of these experiments provide evidence that tryptase-induced mouse brain microvascular endothelial cell activation may be involved in the disruption that occurs in neurodegenerative diseases.

## Abbreviations

BBB: Blood–brain barrier
CNS: Central nervous system
IL-6: Interleukin-6
MC: Mast cell
MMP2: Matrix metalloproteinase 2
MMP9: Matrix metalloproteinase 9
PAR-2: Protease-activated receptor 2
POCD: Postoperative cognitive dysfunction
TJP: Tight junction protein
TLR4: Toll-like receptor-4
TNF-α: Tumour necrosis factor-α
CCK-8: Cell counting Kit-8
ELISA: Enzyme-linked immunosorbent assay
FS: FSLLRY-NH2
MAPK: Mitogen-activated protein kinases
ERK: Extracellular regulated protein kinases
NF-kappa B: Nuclear factor kappa B
PBS: Phosphate buffer saline
